# Preparing to fight back: generation and storage of priming compounds

**DOI:** 10.3389/fpls.2014.00295

**Published:** 2014-06-24

**Authors:** Victoria Pastor, Andrea Balmer, Jordi Gamir, Victor Flors, Brigitte Mauch-Mani

**Affiliations:** ^1^Institute of Biology Laboratory of Molecular and Cell Biology, University of NeuchâtelNeuchâtel, Switzerland; ^2^Metabolic Integration and Cell Signaling Group, Plant Physiology Section, Department of CAMN, Universitat Jaume ICastellon, Spain

**Keywords:** priming, β-aminobutyric acid (BABA), metabolites, primary metabolism, amino acids, induced resistance

## Abstract

Immune-stimulated plants are able to respond more rapidly and adequately to various biotic stresses allowing them to efficiently combat an infection. During the priming phase, plant are stimulated in absence of a challenge, and can accumulate and store conjugates or precursors of molecules as well as other compounds that play a role in defense. These molecules can be released during the defensive phase following stress. These metabolites can also participate in the first stages of the stress perception. Here, we report the metabolic changes occuring in primed plants during the priming phase. β-aminobutyric acid (BABA) causes a boost of the primary metabolism through the tricarboxylic acids (TCA) such as citrate, fumarate, (S)-malate and 2-oxoglutarate, and the potentiation of phenylpropanoid biosynthesis and the octodecanoic pathway. On the contrary, *Pseudomonas syringae* pv tomato (*PstAvrRpt2*) represses the same pathways. Both systems used to prime plants share some common signals like the changes in the synthesis of amino acids and the production of SA and its glycosides, as well as IAA. Interestingly, a product of the purine catabolism, xanthosine, was found to accumulate following both BABA- and *PstAvrRpt2*-treatement. The compounds that are strongly affected in this stage are called priming compounds, since their effect on the metabolism of the plant is to induce the production of primed compounds that will help to combat the stress. At the same time, additional identified metabolites suggest the possible defense pathways that plants are using to get ready for the battle.

## Introduction

Plants are generally confined to one location for their whole lifetime and had therefore to develop adaptive and defensive mechanisms against biotic and abiotic stresses that take this limitation into account. The first crucial step for a plant is to recognize that it is attacked (Nimchuk et al., 2003). The speed by which the plant senses and accurately recognizes a specific stress(or) determines how appropriate and successful its reaction will be. Failure to mount a timely or fitting response will, in case of pathogen attack, lead to colonization of the host tissues and hence to disease (Ebel and Cosio, 1994; Jones and Takemoto, 2004). This basal immunity of a plant contributes to slowing down the colonization process but is generally too weak to effectively prevent disease (Nürnberger and Lipka, 2005). The level of basal immunity of a plant, however, can be enhanced through application of appropriate stimuli. This is commonly referred to as induced resistance (IR). Plants have acquired the ability to widely improve their defensive capacity against a broad range of pathogens including viruses, fungi, oomycetes, and bacteria toward which they are genetically speaking susceptible (Durrant and Dong, 2004; Hammerschmidt, 2009). This defense has to be triggered by an inducing treatment. Various such treatments have been shown to successfully induce resistance. They consist among others of an inoculation with pathogens, rhizobacteria or a treatment with defined chemicals and lead to horizontal resistance of the plant against a broad range of pathogenic organisms (van Loon et al., 1998; Oostendorp et al., 2001; Cohen, 2002; Hammerschmidt, 2009). This resistance operates in all plant parts distant from the original locus of inoculation and is therefore called systemic resistance (Durrant and Dong, 2004).

The initial phase of resistance induction, where the plant is preparing for a future attack but has not yet been challenged by a pathogen is called the priming phase (Conrath et al., 2002). Priming leads to a physiological state in which a plant responds faster and/or more accurately to an attack (Prime-A-Plant Group et al., 2006). This phase lies between the perception of the priming cue and the first exposure to a future stress. During this time slot the plant has to generate and store information that will enable it to deploy this faster and/or more accurate response to stress. How a plant senses and translates a priming cue is not known, however, there is a lot of information on what stimuli successfully induce the priming state. They reach from avirulent pathogens, insect pests, microbe- and host-derived molecules, synthetic substances up to metabolic disturbances of the plant and abiotic stressors (Prime-A-Plant Group et al., 2006). This large diversity in priming triggers suggests that multiple approaches could lead to the induction of the priming state. There is evidence that some of these primary stimuli target epigenetic mechanisms (Bruce et al., 2007). It had been suggested that histone modification and histone replacement could take place at the onset of priming (van den Burg and Takken, 2009). In the meantime it has been shown that the induction of JA-dependent defenses through infection *with Alternaria brassicicola* and *Botrytis cinerea* or by JA itself leads to histone methylation in the promotors of JA-inducible genes (Berr et al., 2010). In rice, *JMJ70*5 codes for a histone lysine demethylase that is induced upon pathogen infection. Overexpression of this histone demethylase results in an increase of resistance against bacterial blight (Li et al., 2013). An association of SA-dependent defenses with NPR1-dependent post-translational modifications of histone tails in promotors of genes coding for defense-related TFs has also been reported (Jaskiewicz et al., 2011). All this hints to an implication of epigenetic mechanisms right at the onset of priming.

There is not much information available on the actual priming phase that follows such epigenetic changes. Possible mechanisms underlying the actual priming have recently been reviewed (Pastor et al., 2013a). The earlier proposition that the priming process is associated with an accumulation of inactive protein kinases has been substantiated by the fact that treatment with the SAR inducer benzo(1,2,3) thiadiazole-7-carbothioic acid S-methyl ester (BTH) leads to an accumulation of inactive MAP kinase 3 (MPK3) and MPK6 (Beckers et al., 2009). Induction of resistance by β-aminobutyric acid (BABA) and the concomitant priming of SA-dependent defenses correlates with a higher expression of SA-regulatory transcription factor (TF) genes and priming with rhizobacteria (WCS417r) leads to an enhanced expression of jasmonic acid (JA)-TF regulatory genes (Van der Ent et al., 2009). The mentioned inactive protein kinases could then be rapidly activated upon stress exposure and the availability of the TF allow for a more effective defense signaling. Hence, both mechanisms could possibly contribute to a faster reaction of the plant.

Plants have also been shown to accumulate inactive defense-metabolite conjugates in their vacuoles that could be released upon attack. Well-known examples are the phytoanticipins glycosinolates and benzoxazinoids, both released into an active form upon hydrolysation with glucosidases (Morant et al., 2008). Plant hormone conjugates could also play a role in priming. Methylated and glucosylated abscisic acid (ABA)-conjugates are mainly stored in vacuoles (Kaiser et al., 1985) and free ABA is then released upon contact with apoplastic esterases (Sauter et al., 2002). During infection of tobacco plants, the induced salicylic acid (SA) is partially metabolized into SA 2-O- beta-D-glucose (SAG) by a SA glucosyltransferase (SAGT) (Edwards, 1994; Lee and Raskin, 1998, 1999; Dean and Mills, 2004; Dean et al., 2005; Song, 2006). Such bound SA could be rapidly liberated through the action of a beta-glucosidase (Seo et al., 1995) when the plant is challenged (Dean et al., 2005). Interestingly, an Arabidopsis mutant impaired in SAG and SEG (*ugt74f1*) is more susceptible to infection by *Pseudomonas syringae* pv tomato (*Pst*) than its wild type counterpart (Boachon et al., 2014) and is partially blocked in BABA-IR against *Pst* (Flors and Mauch-Mani, unpublished). In maize plants, it was shown that following resistance induction with the hemibiotrophic fungus *Colletotrichum graminicola*, the plants accumulate an array of metabolites comprising, besides the well-described benzoxazinoids, molecules such as apigenin, genkwanin, and chlorogenic acid with known fungicidal effects. Interestingly, the reaction of maize to priming treatment was shown to be organ-specific (Balmer et al., 2012; Balmer and Mauch-Mani, 2013).

The specific events targeted by priming (the primed mechanisms) depend on the attacker or stress to be countered. Among these early events are the control of stomatal closure in defense against pathogens using stomata as their entry point into the plant (Jakab et al., 2005), the generation of reactive oxygen species (Moller et al., 2007; Trouvelot et al., 2008; Pastor et al., 2013b), interference with effector-triggered susceptibility (Jones and Dangl, 2006) or callose deposition at the attempted points of entry (Garcia-Andrade et al., 2011). At later time points, priming can influence hormonal signaling pathways such SA-, JA, ABA-, and ET signaling (Ton and Mauch-Mani, 2004; Flors et al., 2008; Jung et al., 2009; Van der Ent et al., 2009; Conrath, 2011; Rasmann et al., 2012). While interactions with biotrophic pathogens are generally controlled via the SA pathway, necrotrophic pathogens and insects are rather contained by mechanisms depending on JA/ET signaling (Glazebrook, 2005). The ABA pathway on the other hand plays an important role in protective mechanisms involving callose deposition or stomatal closure (Ton and Mauch-Mani, 2004; Jakab et al., 2005; Hamiduzzaman et al., 2005; Ton et al., 2009).

More sensitive chromatographic techniques make it now possible to identify metabolites that accumulate during the priming phase as well as in the phase following challenge by a stressor (priming vs. primed metabolites). Several secondary metabolites that mediate priming have recently been identified. Among them figure azelaic acid (Jung et al., 2009), imprimatins (Noutoshi et al., 2012), indol-3-carboxilic acid (I3CA; Gamir et al., 2012), pipecolic acid (Návarová et al., 2012) or galacturonic acid and hypoxanthine (Gamir et al., 2014).

The aim of the present study was to establish a metabolomic profiling of the priming phase of *Arabidopsis thaliana* following induction of priming by two different priming cues, i.e., priming by inoculation with avirulent Pseudomonas and chemical priming by soil-drench with BABA. The ultimate goal was to determine whether different priming stimulus can prepare the plant during the priming phase activating common/different metabolic pathways.

## Materials and methods

### Biological material

Arabidopsis accession Col-0 seeds were germinated in soil and maintained at 21°C day/18°C night, with 9 h of light (125 μE m^−2^ s^−1^) and 60% of relative humidity. One week after germination seedlings were individually transferred to 33 mL Jiffy pellets and kept in the same conditions until the treatments. The experiments were performed with 4–5 weeks-old plants. The avirulent strain *Pseudomonas syringae* pv tomato DC3000 (*PstAvrRpt2*) was grown overnight in liquid medium King B with antibiotics rifampicin (50 μg × mL^−1^) and kanamicin (25 μg × mL^−1^) for selection.

### Plant treatments, sampling, and metabolite extraction

Col-0 plants were soil-drenched with 250 μM of BABA (as a final concentration) or water (as control). For the avirulent strain *PstAvrRpt2*, 2–3 leaves of plants of the same age as for the BABA treatments (4–5 weeks old) were dip-inoculated in the bacterial suspension of 10^9^ colony-forming units mL^−1^ in 10 mM of MgSO_4_ and 0.01% of Silwet L-77 for 4 s, or in the same solution without bacteria (as a control). Samples were taken at 24 and 48 hpt, 3–4 h after the onset of the light phase. The material was lyophilized, extracted in MeOH 10% and further processed as described in Gamir et al. (2012).

To ensure that the plants used for the experiments were primed, for each experiment sample plants were challenged with virulent *Pseudomonas syringae* pv tomato (*Pst*) at a concentration of 10^5^ colony-forming units (cfu). mL^−1^ as described previously (Slaughter et al., 2012). The disease phenotype was assessed 5 days after challenge and expressed as number of symptomatic leaves.

### LC-ESI full scan mass spectrometry (Q-TOF instrument)

Metabolome analysis was performed using an Acquity UPLC system (Waters, Mildford, MA, USA) interfaced to hybrid quadrupole time-of-flight (QTOF MS Premier). The LC separation was performed by HPLC SunFire C18 analytical column, 5 μm particle size, 2.1 × 100 mm (Waters). Analytes were eluted with a gradient of methanol and water containing 0.01% HCOOH. Chromatographic conditions and QTOF MS parameters were followed as described in Gamir et al. (2012).

### Full scan data analysis

Raw data obtained from Masslynx software was transformed to .CDF using Databrigde provided by Masslynx package. The .CDF data was process with R for statistical computing using XCMS package for relative quantification (Smith et al., 2006). For Principal Component Analysis (PCA), heatmap construction and clustering of metabolite the software MarVis Filter and MarVis cluster (http://marvis.gobics.de/; Kaever et al., 2012) were used. The most prominently induced compounds and others shown in text were subjected to identification (Table 1) and the relative intensity of accumulation was analyzed using a paired non-parametric Wilcoxon Mann–Whitney test to confirm a significant difference between the samples (*P* < 0.05).

**Table 1:** **List of compounds identified by exact mass and fragmentation spectrum**.

**Compound**	**Mass (neutral)**	**Fragments**	**ESI**	**RT (min)**
Citrate/isocitrate	192.027	111.010, 87.011	–	2.81
Fumarate	116.013	71.0148	–	3.06
S-Malate	134.022	115.005, 89.027	–	1.81
Succinate	118.029	73.032	–	1.43
2-Oxoglutarate	146.023	101.026, 73.016	–	4.30
9(S)-HPOT/2(R)-HPOT	310.214	309.106, 291.185	–	12.90
13(S)-HOT	294.184	236.106, 221.157	–	12.40
OPC-8:0/9(S)-HOT	294.217	275.202, 96.962	–	13.18
13 (S)-HODE	296.234	71.018	–	13.37
Sinapate	224.069	208.03, 93.037	–	8.50
Sinapoyl malate	340.0806	223.063, 133.015	–	9.41
1-O-Sinapoyl-β-D-glucose	386.122	223.065, 190.026	–	3.48
Xanthosine	284.077	151.029	–	1.81
Guanosine	283.093	133.016	–	1.52

9(S)-HPOT, (10E,12Z,15Z)-(9S)-9-Hydroperoxyoctadeca-10,12,15-trienoic acid; 2(R)-HPOT, (2R)-(9Z,12Z,15Z)-2-Hydroperoxyoctadecatri-9,12,15-enoic acid; 13 (S)-HOT, (9Z,11E,15Z)-(13S)-Hydroxyoctadeca-9,11,15-trienoate; OPC-8:0, 8-[(1R,2R)-3-Oxo-2-{(Z)-pent-2-enyl}cyclopentyl]octanoate; 9(S)-HOT, (9S)-(10E,12Z,15Z)-9-Hydroxyoctadecatri-10,12,15-enoic acid; 13 (S)-HODE, (13S)-Hydroxyoctadecadienoic acid.

## Results

### Different priming stimuli impose subtle metabolic changes in the absence of challenge

**Figure 1:**
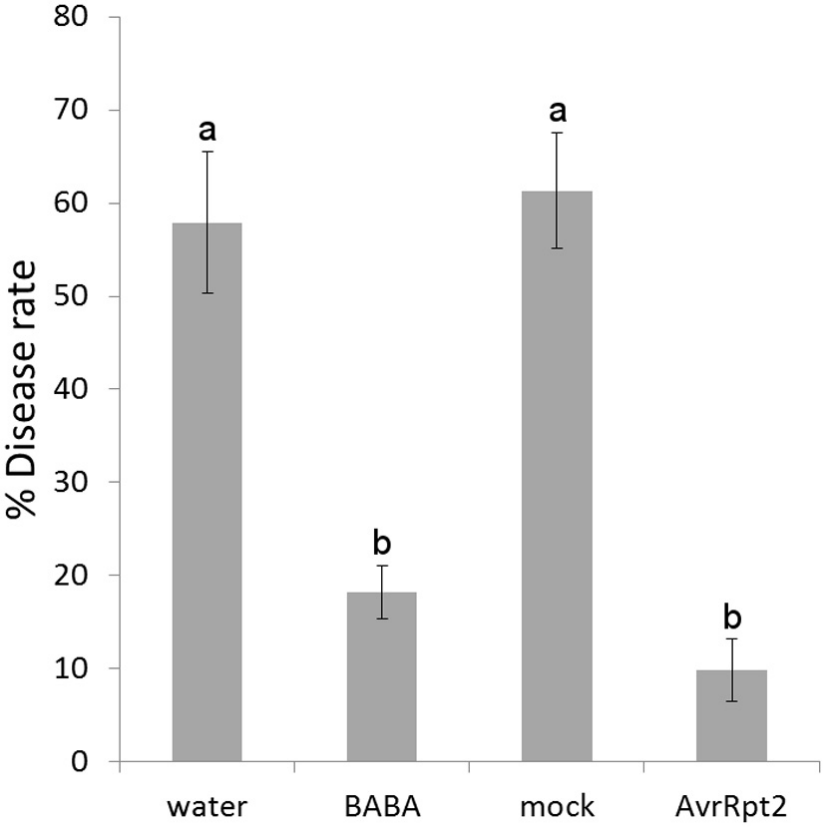
**Disease symptoms in Col-0 plants**. Arabidopsis plants were inoculated with a bacterial suspension of *Pst* at 10^5^ colony-forming units (cfu). mL^−1^. Disease symptoms were determined after 5 days of inoculation and quantified as the proportion of leaves with symptoms. The values are means of the percentage of diseased leaves per plant ± SD. Data presented are from a representative experiment that was repeated for every experiment with similar results. Different letters indicate statistically significant differences between control and infected plants (*p* < 0.05, *n* = 20–25).

The primed state can be induced by various agents. In order to differentiate between chemical and biological induction, BABA was used as a chemical inducer (Zimmerli et al., 2000; Jakab et al., 2001; Ton and Mauch-Mani, 2004) and the avirulent bacterium *Pseudomonas syringae* carrying the avirulence gene *AvrRpt2* (*PstAvrRpt2*; Mudgett and Staskawicz, 1999) as a biological inducer of resistance. Since metabolites are the final products resulting from metabolic pathways, the present work aims to identify, the main compounds that could act as signals during the priming phase and the involved pathways. It is presumed that at first contact with BABA or *PstAvrRpt2* a plant response is initiated that will allow it to get ready to combat further stresses. To ensure that the plants used in our experiments were primed, for each experiment sample plants were challenged and the resulting disease phenotype was assessed (Figure 1).

**Figure 2:**
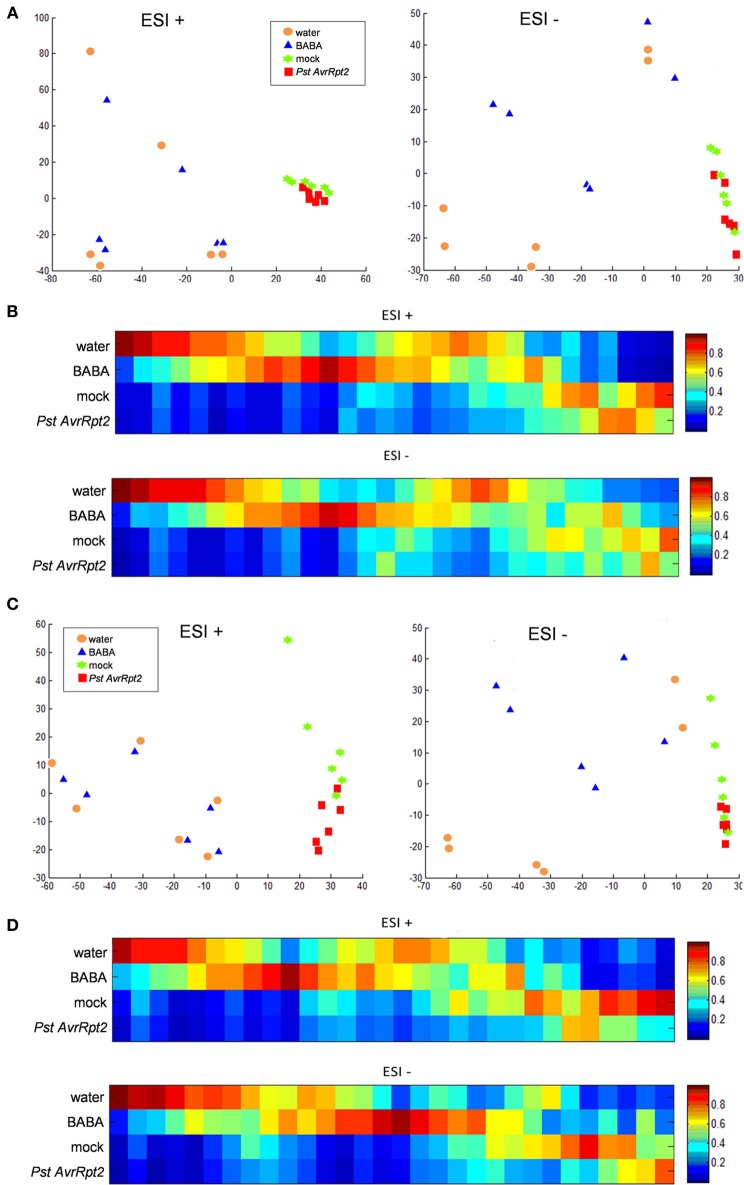
**Principal component analysis (PCA) and cluster plots comparing the four groups generated from the major sources of variable signals obtained in ESI+ and ESI− by non-targeted analysis by HPLC-QTOF MS of the four groups water, BABA, mock, and *PstAvrRpt2***. The PCA and cluster analyses were performed using Marvis Filter and Cluster packages, following a Kruskal–Wallys test (*p* < 0.05). **(A)** PCA analysis score plot after 24 hpt. **(B)** Cluster plot of main compounds of the four groups after 24 hpt. **(C)** PCA analysis score plot at 48 hpt. **(D)** Cluster plot of significant compounds after 48 hpt.

To this end *Arabidopsis* Col-0 plants were treated with BABA and *PstAvrRpt2* with water- and mock-treated plants as control, respectively. To gain a global overview of the metabolite balance during the priming phase, i.e., the 48-h time interval before the stress is usually applied (Pastor et al., 2013a and the references within), a metabolic analysis with UPLC coupled to Q-TOF mass spectrometry (quadrupole-time of flight mass spectrometer) was performed. Metabolic and bioinformatic analysis of the signals obtained in positive and negative electrospray ionization (ESI) were done according to the methods of Fernie et al. (2011), Kaever et al. (2012), and Gamir et al. (2014). When comparing the four groups (Figure 2; water, BABA, mock, and *PstAvrRpt2*) the principal component analysis (PCA) shows the global behavior of the signals in both modes of ESI at 24 hpt and 48 hpt. BABA and *PstAvrRpt2* both induce qualitative different metabolites, easily visualized in the cluster realized by the Marvis Cluster software. This might point to at least partially different mechanisms of priming action for BABA and *PstAvrRpt2*.

Signals found in BABA-treated plants do not overlap but remain close to control plants. This confirms the previous view that priming does not lead to major changes in a plant (Conrath, 2009). The fact that in positive mode the signals are closer than in negative mode means that in general, the compounds that are negatively ionized could be more important in the establishment of priming, since they present a higher difference in respect to the control. On the contrary, inoculation with avirulent bacteria shows that the action of bacteria is slower than BABA. Separation between bacteria and mock treatment becomes visible only after 48 hpt.

The differences between treatments was visualized through a supervised heatmap analysis using the signals that showed a significant difference (Kruskal–Wallis test *P* < 0.05). At both 24 and 48 hpt, there is more dynamism in the group of compounds stemming from BABA-treated plants than from *PstAvrRpt2*-inoculated plants. However, when clustering water vs. BABA and mock vs *PstAvrRpt2* (Figures S1, S2) the differences between treated and not treated plants are more visible. All together BABA-treatment leads to a more rapid induction of the priming phase than inoculation with bacteria does.

### Chemical and biological priming targets primary metabolism during priming phase: the role of carboxylic acids

**Figure 3:**
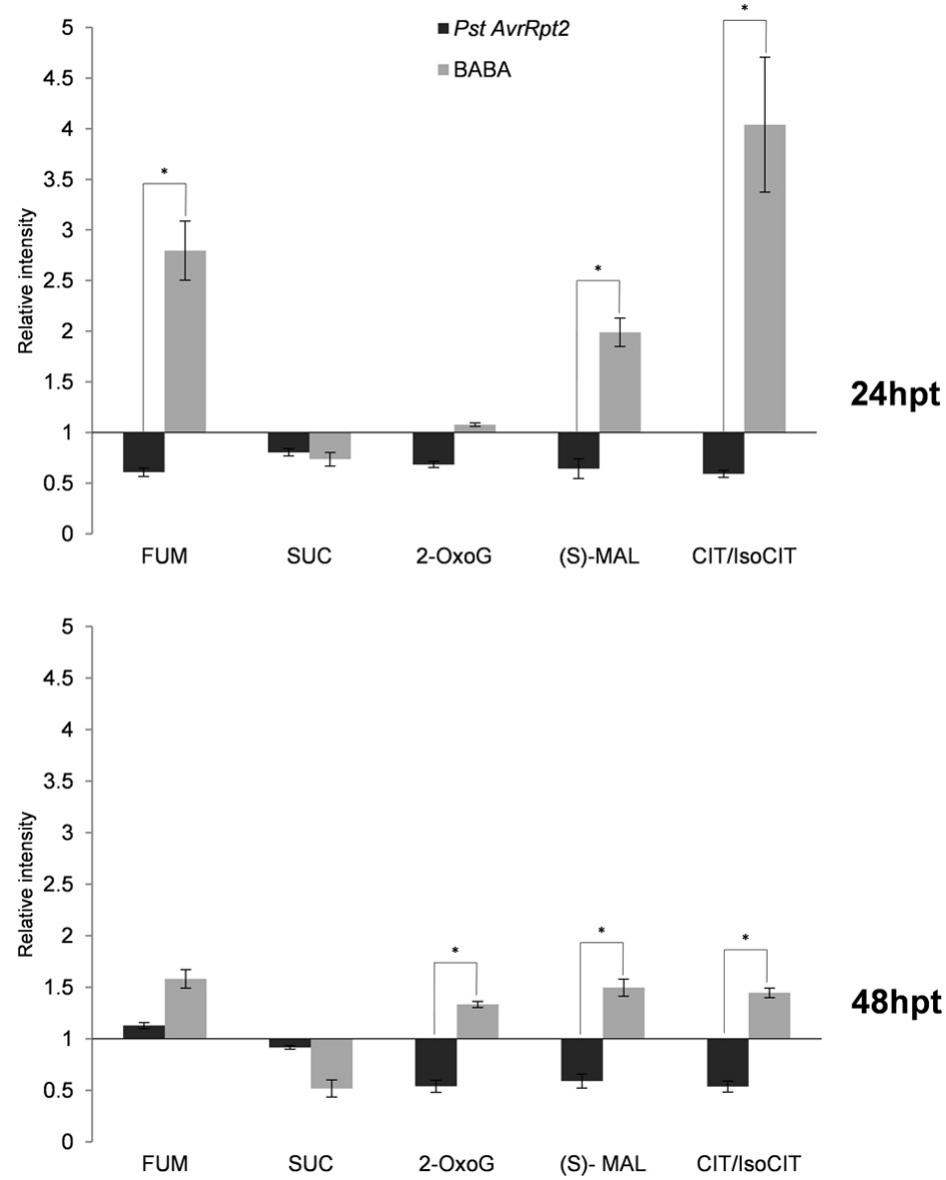
**Profile of compounds related to the tricarboxylic acid cycle**. Four five-week-old Col-0 plants were processed for relative quantification after 24 and 48 hpt. The concentration of metabolites was obtained by normalizing the chromatographic area of each compound to the dry weight of the sample. The bars show the relative accumulation (relative intensity) of each compound in respect to its control, BABA vs. water, *PstAvrRpt2* vs. mock. Asterisks represent statistically significant differences (Wilcoxon Mann–Whitney test, *p* < 0.05, *n* = 6). Data represent the average of three independent experiments.

Priming is a horizontal phenomenon potentiating basal defenses and further layers of defense in case basal defense is defective (Pastor et al., 2013b). The plant is put in a state of alert allowing a wide elasticity in the use of defenses. Therefore, priming metabolites might be very primary compounds that can be channeled to any direction in order to rapidly strengthen the defensive state of the plant. To determine the most relevant compounds in priming, the most strongly induced signals in the clusters were identified by exact mass, fragmentation spectrum and the retention time of the fragments using the Metlin (http://metlin.scripts.edu) and MassBank (http://www.massbank.jp) databases, and, if necessary, the Kegg and Aracyc databases (http://www.genome.jp/kegg/; http://pathway.gramene.org/gramene/aracyc.shtml). Interestingly, the most induced compounds by BABA belong to the tricarboxylic acid cycle (TCA). These compounds accumulated to a lesser degree following *PstAvrRpt2* infection (Figure 3). Citrate/isocitrate production was strongly induced by BABA at 24 and 48 hpt and, to a lesser extent, also a (S)-Malate, 2-Oxoglutarate and fumarate. Interestingly, *PstAvrRpt2* repressed the accumulation of all these compounds. Nevertheless, succinate was less present in plants treated by both types of priming. Hence, BABA-induced priming functions through the potentiation of the TCA cycle, while *PstAvrRpt2*-induced priming acts differently. All this suggests that the primary metabolism plays an important role in priming for defenses, and is a key point in defining the mechanisms operating in compatible interactions.

### Chemical and biological priming targets primary metabolism during priming phase: changes in amino acids

The compounds involved in TCA flux are the origin for different amino acids pathways. Defense responses against pathogens and insects induce antimicrobial and signaling compounds such as glucosinolates and their breakdown products (Barth and Jander, 2006; Bednarek et al., 2009), or JA-Ile (Fonseca et al., 2009). These secondary metabolites are derivatives or conjugates of amino acids. The balance of amino acids has a major impact on plant defense and priming (Liu et al., 2010; Singh et al., 2010; Návarová et al., 2012; Zeier, 2013; Gamir et al., 2014). In order to know whether these metabolites play a role during the priming phase after BABA and *PstAvrRpt2* treatment, the amino acids were identified using a library of amino acids set up with standards (Gamir et al., 2014).

**Figure 4:**
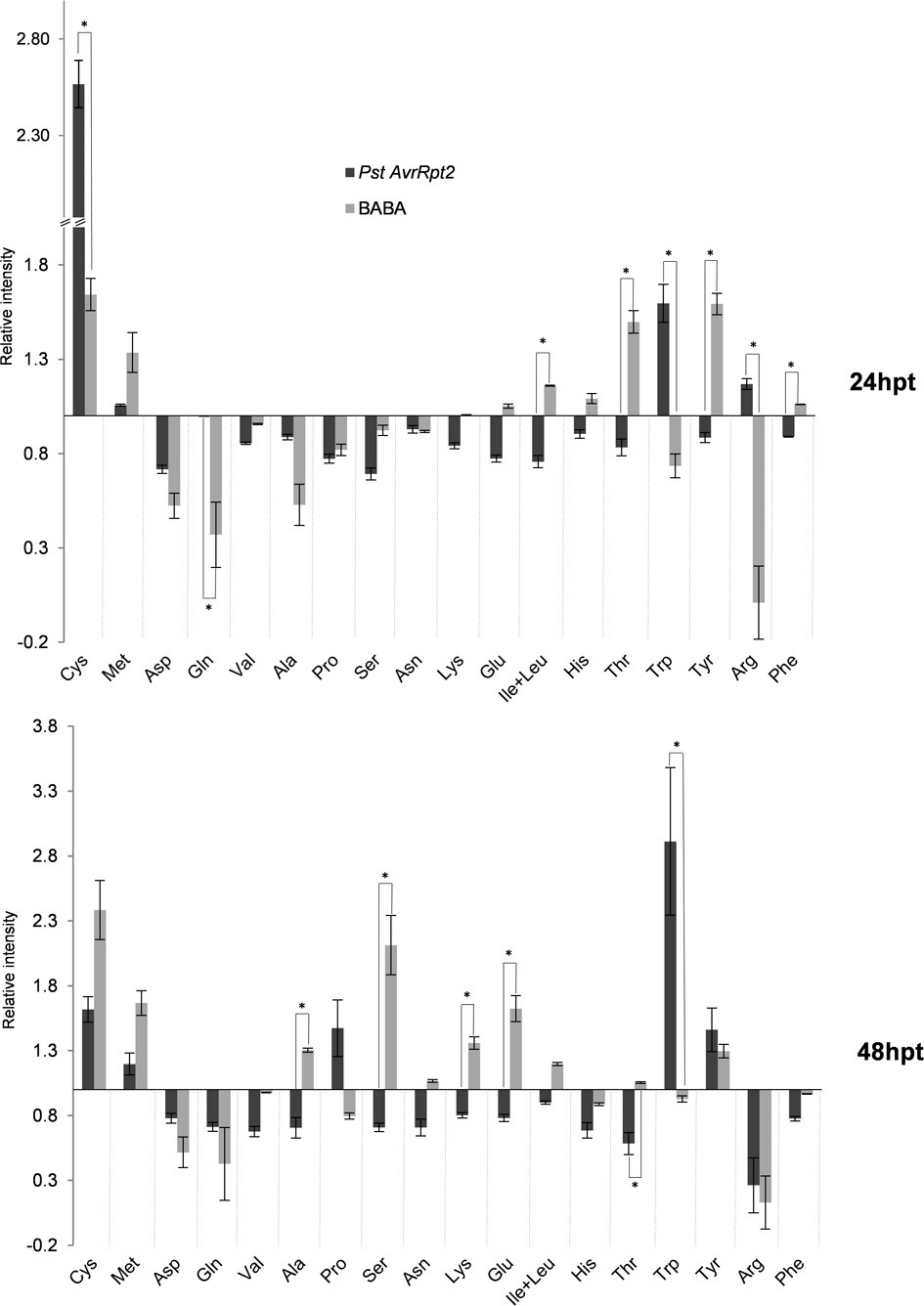
**Amino acid profile of plants primed with BABA and *PstAvrRpt2*, respectively**. The abundance of each amino acid was calculated as described for Figure 2. Col-0 plants, 4–5 week-old, were harvested at 24 and 48 hpt. Asterisks represent statistically significant differences (Wilcoxon Mann–Whitney test, *p* < 0.05, *n* = 6). Data represent average of three independent experiments.

**Figure 5:**
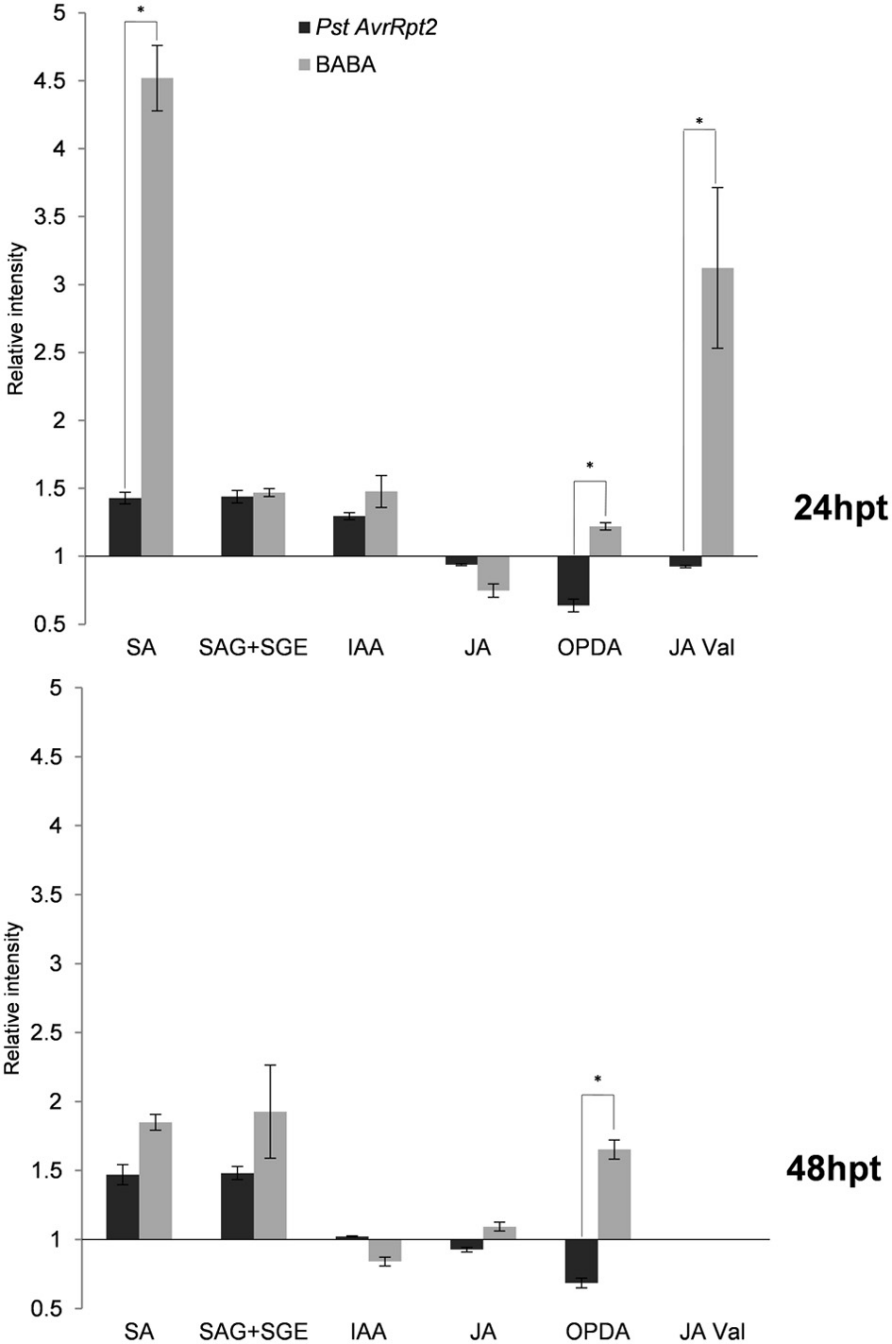
**Levels of hormones and conjugates after 24 and 48 hpt**. The abundance of every compound was calculated as in Figure 2. Asterisks represent statistically significant differences (Wilcoxon Mann–Whitney test, *p* < 0.05, *n* = 6). Data represent average of three independent experiments.

After 24 hpt (Figure 4) BABA-treated plants accumulated Cys, Met, Glu, Ile+Leu (no distinguishable in our chromatographic conditions), His, Thr, Tyr, and Phe. At the same time, the plants primed by avirulent bacteria produced more Cys, Met, and Trp. At the later time point (48 hpt; Figure 5), the levels of Cys, Met, Glu, and Ile+Leu remained high in BABA-treated plants, Thr and Tyr (these last two in lower amounts), but His and Phe were repressed. Other amino acids like Ala, Ser, Asn, and Lys accumulated at this later time point following BABA-priming. On the other hand, plants incoculated with *PstAvrRpt2* kept accumulating Cys, Met, and Trp (this one in higher levels that at 24 hpt), but showed a reduced accumulation of Arg, Pro, and Tyr however were induced at this time point.

Asp, Gln, and Val are less abundant in all time points, independently of the treatment, while Cys, Met, and later, Tyr, were always accumulating under priming conditions. In general, at the beginning, there was depletion in amino acid accumulation during the priming phase, and this effect was more pronounced at 24 hpt.

Thus, the priming state leads to a drop in amino acids during the first hours, and Cys, Met, and Tyr are “priming amino acids” preparing the plant for primed defenses.

### Priming of secondary metabolites

**Figure 6:**
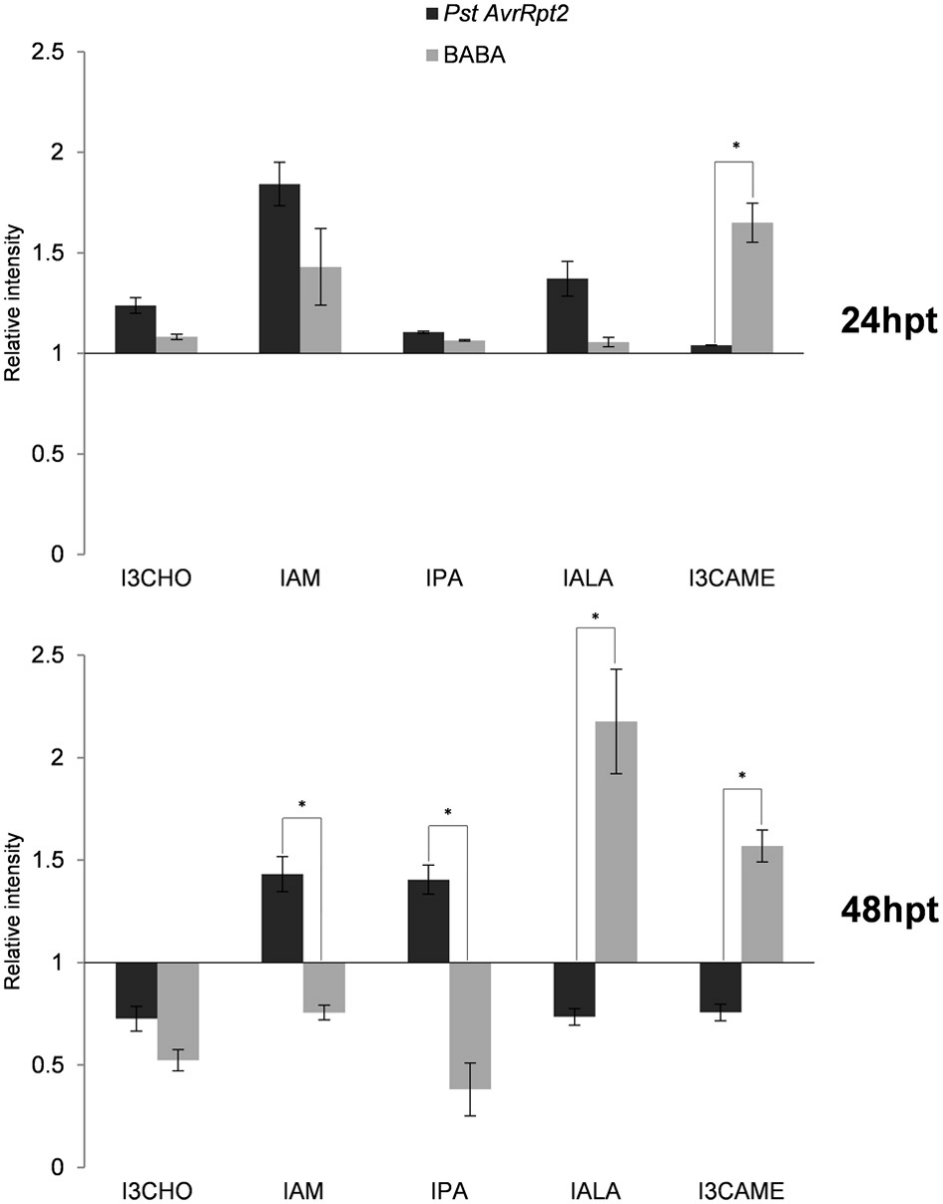
**Profile of indolic compounds during the priming phase**. The experimental conditions and quantification of the levels were calculated as indicated in Figure 2. Asterisks represent statistically significant differences (Wilcoxon Mann–Whitney test, *p* < 0.05, *n* = 6). Data represent average of three independent experiments.

Since the primary metabolism seems to play a central role in the establishment of priming, the contribution of the secondary metabolism was also investigated. Using the library of metabolites (see above), some hormones (Figure 5) and indolic compounds (Figure 6) participating in priming were identified.

Free salicylic acid (SA) and glucosides of SA (SAG+SGE) accumulated in both time points and the induction was higher by BABA than avirulent bacteria. Indole-3-acetic acid (IAA) showed higher levels at 24 hpt but decreased afterwards and was repressed by BABA. Additional indolic compounds have been identified, among them indole-3-carboxaldehyde (I3CHO), indole-3-acetamide (IAM), indol-3-pyruvic acid (IPA), indole-3-acetyl-L-Ala (IALA), and indole-3-carboxylic acid methyl ester (I3CAME). At 24 hpt all of them showed elevated levels in comparison to the controls. Interestingly, IALA was significantly more present in *PstAvrRpt2*- treated than in BABA-treated plants. After 48 hpt, the situation was different, since in BABA-treated plants the levels of IALA remained elevated but in *PstAvrRpt2*-inoculated plants the accumulation of this conjugate was repressed. In general, *PstAvrRpt2* treatment represses the accumulation of most these indolic compounds after 48 hpt. When this is not the case, then BABA-treatment leads to a repression of these compounds.

JA levels were repressed at 24 hpt confirming the well-known crosstalk already between SA and JA (Koornneef and Pieterse, 2008). At 48 hpt JA starts to accumulate in BABA-treated plants. OPDA was consistently accumulating over time in BABA-treated plants and depleted by *PstAvrRpt2* treatment in both time points.

**Figure 7:**
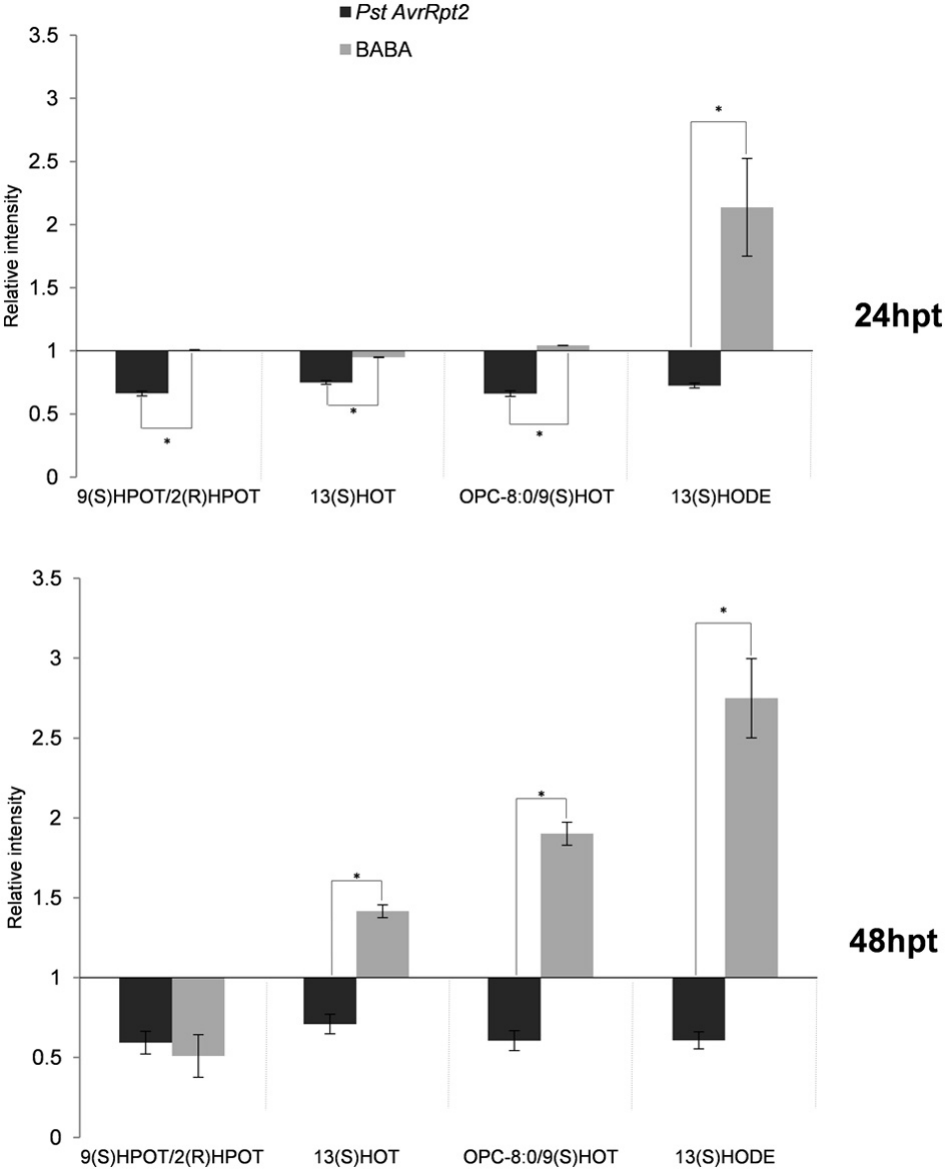
**Fatty acid production in priming**. The experimental conditions and quantification were performed as explained above (Figure 2). Asterisks represent statistically significant differences (Wilcoxon Mann–Whitney test, *p* < 0.05, *n* = 6). Data represent average of three independent experiments.

Interestingly, Val conjugated to JA (JA-Val) showed a strong accumulation upon BABA treatment and the opposite effect resulted from *PstAvrRpt2* treatment at 24 hpt. Using exact mass, fragment spectrum and retention time of fragments, some components of the α-linolenic and linoleic pathways (octadecanoid pathway) were detected, although in our experimental conditions it was not possible to differentiate between some lipid derivatives due to the similarity between fragments (Figure 7). These compounds are described in Table 1. All of them are precursors of OPDA and JA. The bacteria repressed the α-linolenic pathway as well as some compound from the linoleic pathway, such as 13 (S)-HODE. After BABA treatment, accumulation was only seen after 48 h except for 9-(S) HPOT/2(R)-HPOT, which accumulated to a lesser extent.

**Figure 8:**
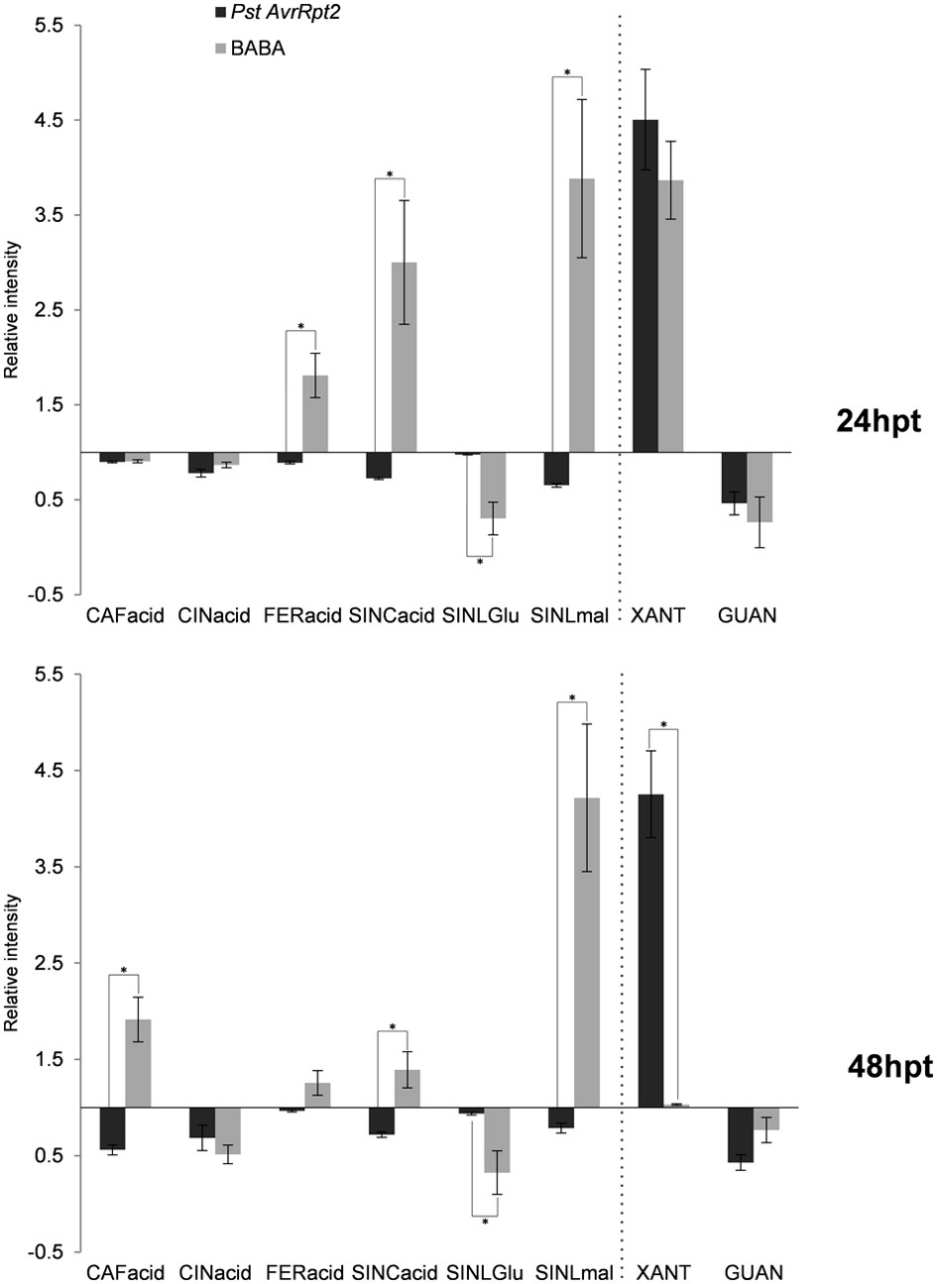
**Phenylpropanoid biosynthesis and purine catabolism compounds in priming**. The experimental conditions and quantification were performed as explained above (Figure 2). Asterisks represent statistically significant differences (Wilcoxon Mann–Whitney test, *p* < 0.05, *n* = 6). Data represent average of three independent experiments.

Caffeic, cinnamic and ferulic acid were identified using the library of standards (Gamir et al., 2014). At 24 hpt only ferulic acid showed an increase and only upon BABA treatment. At 48 hpt caffeic acid accumulated in BABA-treated plants and the levels of ferulic acid remained as at 24 hpt. The bacteria induced the accumulation of cinnamic acid within 48 hpt. All these compounds are related to phenylpropanoid biosynthesis pathway (Figure 8). Additional compounds belonging to this pathway have been identified by exact mass like sinapic acid, 1-O-sinapoyl-β-D-glucose and sinapoyl malate. As shown in Figure 8, BABA strongly stimulated the production of sinapic acid and its ester, sinapoyl malate. The glycosylated intermediate compound (1-O-sinapoyl-β-D-glucose) accumulated to a lesser extent following the treatment suggesting that the glycosylated form is feeding into the production of the ester (Milkowski and Strack, 2010).

### Involvement of purines?

Interestingly, an additional compound, xanthosine, was induced by both types of priming. This nucleoside was strongly induced, almost to the same levels as citrate and the levels remained high during the entire priming phase following inoculation with the bacteria (Figure 8). The accumulation of xanthosine was inversely correlated to the one of guanosine. Both compounds play role in purine catabolism, where the amino group of guanosine is released by guanosine deaminase to produce xanthosine (Dahncke and Witte, 2013).

## Discussion

It is crucial for the survival plants to sense changes taking place in their natural environment. Their cells have to adapt the composition and the levels of metabolites to these changes and if the natural balance of metabolites is disturbed, the plant can answer with massive changes at the transcriptional level to recover the equilibrium (Katagiri, 2004; Kresnowati et al., 2006). An adaptation to such environmental changes is facilitated when the plant has been primed to do so. Priming has been described as a sensitized state in which plants can react more adequately to combat stresses. In a primed plant, the speed and the strength of the answer to a given stress is improved, hence, the primed plant survives better and with less damage. The mechanisms underlying priming are still under discussion. The strategies used by primed plants comprise both, relative early as well as long lasting responses (Pastor et al., 2013a). The latter ones have even been shown to be transferred to the offspring (Luna et al., 2012; Rasmann et al., 2012; Slaughter et al., 2012). However, what is happening in a plant after the perception of a priming cue but prior to encountering the stress remains to be determined. The aim of the present work was to acquire an overview of the metabolites that act as priming compounds and the pathways implicated in the priming phase following treatment of the plants with BABA as a chemical and *PstAvrRpt2* as a biological priming agent. Given the nature of priming, i.e., the rapid reaction against any type of stress (biotic and abiotic), the compounds accumulating before challenge with an actual stressor are expected to belong to the very early phase in the chain of events leading to the biochemical synthesis of secondary metabolites.

The comparison of the effect of the priming treatments (BABA and *PstAvrRpt2*) and their respective control treatments (water and mock,) on the plants' metabolome revealed a different dynamism when comparing BABA- and *PstAvrRpt2*-primed plants. BABA, as an easily water-soluble chemical, is taken up and distributed rapidly in the plant and might therefore lead to faster changes in the metabolome. On the opposite, the bacteria use quorum sensing to organize and coordinate the interaction between themselves and with their host. *PstAvrRpt2* might need more time to induce changes in the plant since the bacteria have first to multiply in order to colonize the tissues (Miller and Bassler, 2001; Schikora et al., 2011).

The metabolites undergoing the most pronounced changes in BABA-treated plants belong to the tricarboxylic acid (TCA) cycle. Citrate/isocitrate (indistinguishable in our chromatographic conditions), show the highest accumulation followed by other compounds of the cycle. Only the levels of succinate are reduced by BABA. On the opposite, *PstAvrRpt2*-treatment leads to a general repression of the TCA cycle. The primary metabolism appears like a key point in priming and highlights the differences between the two kinds of priming inducers.

It is known that both compatible and incompatible interactions lead to a reduction of photosynthesis (Swarbrick et al., 2006; Berger et al., 2007 and references therein) due to the chlorosis provoked by the infection. The reduction of photosynthesis during the resistance responses is accompanied by an increase of carbohydrates in the apoplast and could act as a signal for repression of photosynthesis. The process needs an income of carbon-skeletons that in turn leads to a reduction in the levels of compounds belonging to the TCA cycle (Chou et al., 2000). On the other hand, BABA seems to help in the accumulation of carboxylic acids, perhaps because the plant can perceive the decrease with no apparent reason (no infection) of amino acids following BABA treatment. This could stimulate the production of carboxylic acids (Bolton, 2009) that in turn are not required for other purposes and can be accumulated in order to fuel other pathways (Bolton, 2009). It still needs to be investigated whether the accumulation triggered by BABA is a consequence of a minor stress caused by the chemical or if it is a more direct action due to its amino acid nature. The response to stress is accompanied by a wide mobilization of the defense system, nevertheless, not all defense mechanism are used by the plant (Galletti et al., 2008; Rayapuram et al., 2008). In any case, plant response to stress is highly energy-consuming and depends on carbon supply to sustain the biosynthesis of defense compounds. The TCA cycle not only provides such carbon structures for the biosynthesis but also participates in ATP generation. The accumulation of these compounds can help to provide the energy or the carbon skeletons for future demands. The major C sink is to Glu and Gln from citrate and 2-oxoglutarate (Gauthier et al., 2010), but here we found that after BABA-application, only Glu was accumulating over the time of the experiment and this coincided with the induction of 2-oxoglutarate. On the contrary, Gln levels remained low following both types of priming. A possible explanation could be the simultaneous accumulation of the nucleoside xanthosine, a product of the catabolism of purines. The N structure of the purine ring comes from the Glu, Asp and Gly (Stasolla et al., 2003), and the levels of all three amino acids go markedly down following BABA- and *PstAvrRpt2*-treatment. The accumulation of xanthosine coincides with the depletion in guanosine. In Arabidopsis, xanthosine is generated from the deamination of guanosine with the help of a specific guanosine deaminase (Dahncke and Witte, 2013). Xanthosine is a precursor of caffeine in some plant species but has not been reported in Arabidopsis (Mohanpuria and Yadav, 2009), and to our knowledge, there is not any direct evidence for a role of this compound in defense. The catabolism of purines ends with the production of CO_2_ and nitrogen. But purines also participate in building other molecules such as DNA, RNA or other secondary metabolites, and significant amounts of xanthosine are used for RNA synthesis (Ashihara, 2012). This again suggests that the metabolism of primed plants is getting ready for a future need for defense compounds or help in the synthesis of nucleic acids (Riegler et al., 2011).

Secondary metabolism is important for the final output of the defense response, which can produce long distance signals or, even, biocidal substances inhibiting the action of pathogens and insects. During the priming phase, the accumulation of SA and its glycosides, as well as a repression in the accumulation of JA is taking place. Interestingly, there is a peak in SA accumulation at 24 hpt in BABA-treated plants, and this accumulation is significantly higher than the one induced by *PstAvrRpt2*. After this time point SA remains at the same level in both types of priming. The levels of SA-glycosides do not change during priming and are potentially ready to release free SA upon encounter with a stress. The accumulation of free and conjugated forms of SA after infection with the pathogen *Pseudomonas syringae* pv tomato DC3000 (*Pst*; Pastor et al., 2012) has been reported, and this accumulation is usually accompanied by the repression of genes in the JA pathway and related genes (Koornneef and Pieterse, 2008; Boachon et al., 2014). The complexity of SA synthesis and signaling makes it difficult to get an exact picture of all the possible effects it might have in the plant (Shah, 2003). On the other hand, although JA levels display a similar profile in BABA- and *PstAvrRpt2*-treated plants, the cyclopentenone ODA shows specific behavior depending of the treatment applied. Despite overlapping gene-induction patterns for JA and OPDA (Stinzi et al., 2001), a distinct role for OPDA in signaling can be expected due to the electrophilic nature of the cyclopentenone ring (Farmer et al., 2003). In our experimental conditions, treatment with *PstAvrRpt2* altered the oxylipin pathway through the repression of several precursors of OPDA and JA, while these compounds accumulated at 48 hpt with BABA. The repression of the octadecanoid acid pathway by the *PstAvrRpt2* correlates with the observed depletion of tricarboxylic acids and the accumulation of tricarboxylic compounds by BABA correlates well with the induction of the same pathway. This points to a connection between these two pathways. Interestingly, there is a significant increase of the JA conjugate JA-Val. The (+)-JA-Ile conjugate has been shown to be the active isomer in the signaling pathway, but there is little information about the role of other JA-amino acid conjugates. JA-Ile and JA-Val accumulate in response to oral secretions of *Manduca sexta* mediated by *JAR6*, a homolog of *JAR1*, in *Nicotiana attenuata*,(Wang et al., 2007). In our experimental conditions, an accumulation of JA-Val was only observed at 24 hpt, and JA-Ile was not detected. It is conceivable that the conjugation with amino acids leads to an increase of the bioactivity of JA, but further studies are needed to get a clear picture about the role of amino acid conjugates in general defense responses.

The influence of the TCA cycle on the biosynthesis of other biomolecules also linked to the phenylpropanoid biosynthesis. BABA treatment led to an accumulation of sinapate and its ester, sinapoyl malate, while *PstAvrRpt2* treatment caused depletion in these two phenylpropanoids. Thus, BABA has a major impact on this pathway that is supplying precursers of lignin (Goujon et al., 2003). BABA has been shown to play a major role in the modification of the plant cell walls after pathogen attack (Ton and Mauch-Mani, 2004), and it is tempting to hypothesize that phenolics and sinapates act as priming compounds facilitating the synthesis of molecules needed for the reinforcement of the cell wall. A similar mechanism could be imagined for IAA in priming the accumulation of indolic compounds, although both BABA and the bacteria lead to similar accumulation of IAA and the profile of the indolic compounds identified is more variable. Tryptophan (Trp) metabolism leads to the synthesis of glucosinolates, phytoalexins and auxins, and their regulation is dependent on a branched network that makes it difficult to obtain a clear profile. The induction of the Trp pathway by the bacteria is followed by the accumulation of two compounds that participate in IAA synthesis and depend on the Trp pathway, namely indole-3-pyruvic acid (IPA) and indole-3-acetamide (IAM; Strader and Bartel, 2008; Sugawara et al., 2009). Here again, the plants respond differentially to the treatments. While the accumulation of IAA via the Trp pathway seems to be more relevant following priming by *PstAvrRpt2*, BABA seems to use a Trp-independent pathway (Cohen et al., 2003) since it leads to a depletion of the levels of Trp. Alternatively, it might consume more Trp to produce IAA as well as others conjugates (IALA) or derivatives of indol-3-carboxylates (I3CAME).

**Figure 9:**
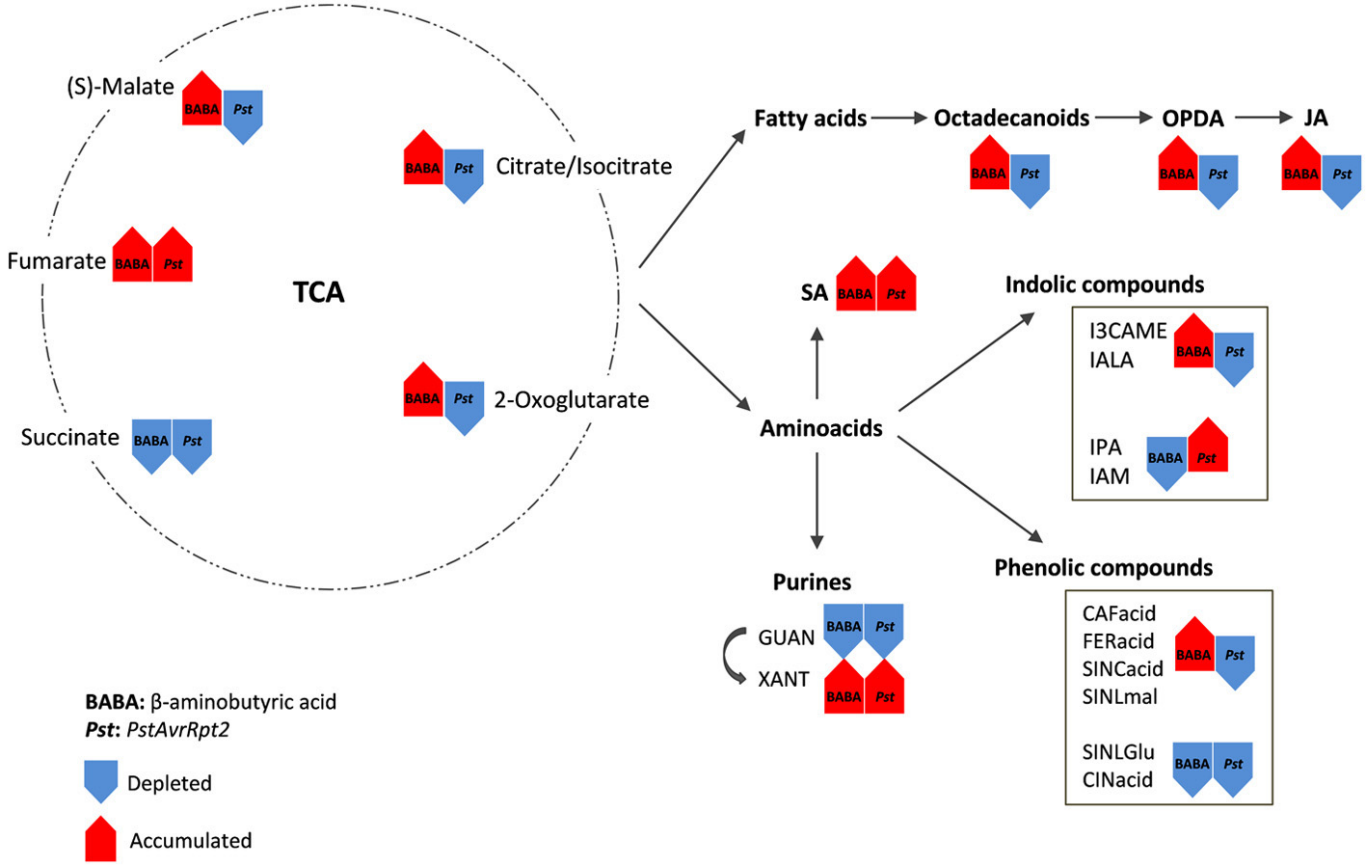
**Principal pathways/compounds that show significant changes after the two priming inducers**. In priming experiments the stress is usually applied after 48 hpt. Rows represent the general changes after this period of time. Red shows the accumulated compounds and blue rows show the depleted compounds/pathways. TCA compounds are the main source for the synthesis of other metabolites playing a major role in defense. These compounds belong to the octadecanoid pathway (fatty acids) and amino acids. Both pathways are divided in subsequent pathways and compounds that are important in defense signaling and decide the final output when the plant meet the stress.

The priming state can be installed by certain compounds that supply the material a plant might need when it encounters a stress. Chemical and biological priming each have a certain specificity that prepares the plant in a different manner (Figure 9), but the final result is the same: the survival of the plant. In nature a plant has to decide rapidly which is the best way to respond to unexpected stress. Priming plants for defense gives them the tools to do so by taking the right decision.

### Conflict of interest statement

The authors declare that the research was conducted in the absence of any commercial or financial relationships that could be construed as a potential conflict of interest.

## Supplementary material

Figure S1 **Principal component analysis (PCA) and cluster plots comparing water and BABA groups generated from the major sources of variable signals obtained in ESI+ and ESI− by non-targeted analysis by HPLC-QTOF MS of the four groups water, BABA, mock, and *PstAvrRpt2***. The PCA and cluster analyses were performed using Marvis Filter and Cluster packages, following a Kruskal–Wallys test (*p* < 0.05). **(A)** PCA analysis score plot after 24 hpt. **(B)** Cluster plot of main compounds of the four groups after 24 hpt. **(C)** PCA analysis score plot at 48 hpt. **(D)** Cluster plot of significant compounds after 48 hpt.

Figure S2 **Principal component analysis (PCA) and cluster plots comparing the mock and *PstAvrRpt2* groups generated from the major sources of variable signals obtained in ESI+ and ESI− by non-targeted analysis by HPLC-QTOF MS of the four groups water, BABA, mock, and *PstAvrRpt2.*** The PCA and cluster analyses were performed using Marvis Filter and Cluster packages, following a Kruskal–Wallys test (*p* < 0.05). **(A)** PCA analysis score plot after 24 hpt. **(B)** Cluster plot of main compounds of the four groups after 24 hpt. **(C)** PCA analysis score plot at 48 hpt. **(D)** Cluster plot of significant compounds after 48 hpt.
