# Supplementary material for: Preparing to fight back: generation and storage of priming compounds
Source: Front Plant Sci. 2014 Jun 24;5:295. doi: 10.3389/fpls.2014.00295 (PMC4068018; doi:10.3389/fpls.2014.00295)
Supplement: Figure S1 — Principal component analysis (PCA) and cluster plots comparing water and BABA groups generated from the major sources of variable signals obtained in ESI+ and ESI− by non-targeted analysis by HPLC-QTOF MS of the four groups water, BABA, mock, and PstAvrRpt2. The PCA and cluster analyses were performed using Marvis Filter and Cluster packages, following a Kruskal–Wallys test (p < 0.05). (A) PCA analysis score plot after 24 hpt. (B) Cluster plot of main compounds of the four groups after 24 hpt. (C) PCA analysis score plot at 48 hpt. (D) Cluster plot of significant compounds after 48 hpt. [file Presentation1.PDF]

Supplementary Figures

Figure S1. Principal component analysis (PCA) and cluster plots comparing water and BABA groups generated from the major sources of variable signals obtained in ESI+ and ESI- by non-targeted analysis by HPLC-QTOF MS of the four groups water, BABA, mock and *PstAvrRpt2*. The PCA and cluster analyses were performed using Marvis Filter and Cluster packages, following a Kruskal-Wallys test ($p < 0.05$). (A) PCA analysis score plot after 24 hpt. (B) Cluster plot of main compounds of the four groups after 24 hpt (C) PCA analysis score plot at 48 hpt (D) Cluster plot of significant compounds after 48 hpt.

Figure S2. Principal component analysis (PCA) and cluster plots comparing the mock and *PstAvrRpt2* groups generated from the major sources of variable signals obtained in ESI+ and ESI- by non-targeted analysis by HPLC-QTOF MS of the four groups water, BABA, mock and *PstAvrRpt2*. The PCA and cluster analyses were performed using Marvis Filter and Cluster packages, following a Kruskal-Wallys test ($p < 0.05$). (A) PCA analysis score plot after 24 hpt. (B) Cluster plot of main compounds of the four groups after 24 hpt (C) PCA analysis score plot at 48 hpt (D) Cluster plot of significant compounds after 48 hpt.

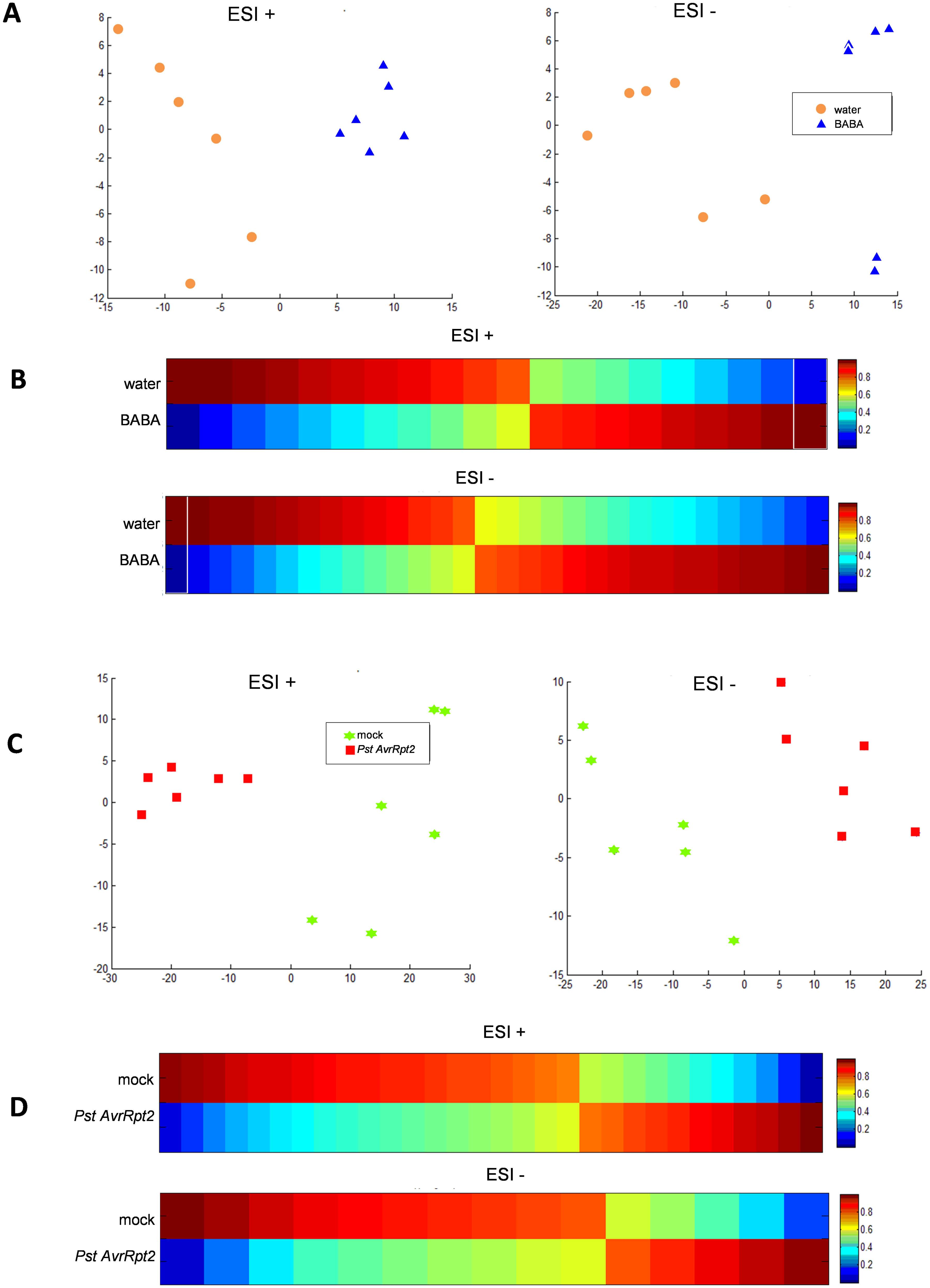
A
ESI +
ESI -
water
BABA
B
ESI +
water
BABA
0.8
0.6
0.4
0.2
ESI -
water
BABA
0.8
0.6
0.4
0.2
C
ESI +
mock
Pst AvrRpt2
ESI -
D
ESI +
mock
Pst AvrRpt2
0.8
0.6
0.4
0.2
ESI -
mock
Pst AvrRpt2
0.8
0.6
0.4
0.2

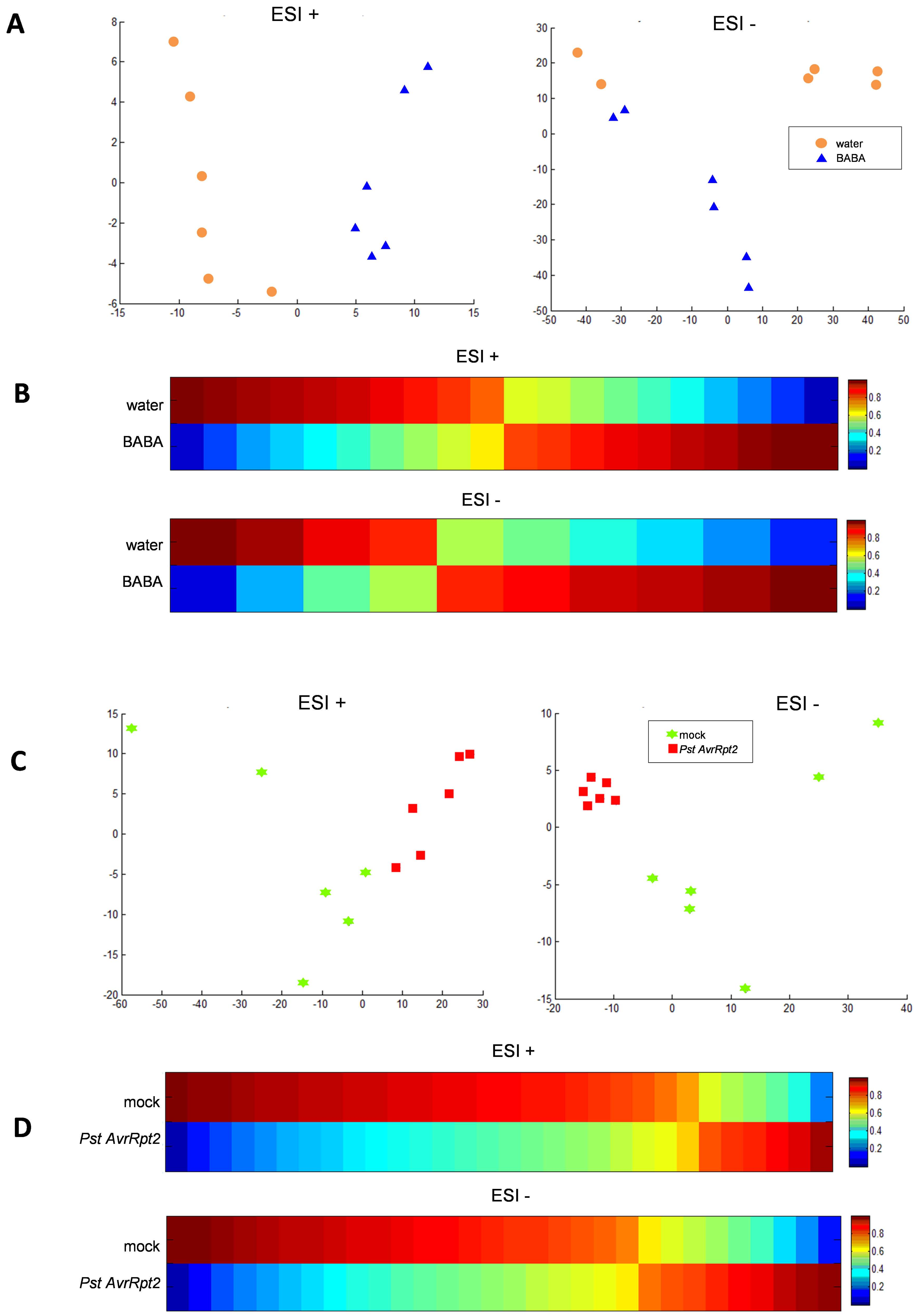
A
ESI +
ESI -
water
BABA
B
ESI +
water
BABA
ESI -
water
BABA
C
ESI +
ESI -
mock
Pst AvrRpt2
D
ESI +
mock
Pst AvrRpt2
ESI -
mock
Pst AvrRpt2
